# Prognostic value of lactate dehydrogenase in patients with uveal melanoma treated with immune checkpoint inhibition

**DOI:** 10.18632/aging.204996

**Published:** 2023-09-05

**Authors:** Xiaocui Liang, Shan Zhou, Zefeng Xiao

**Affiliations:** 1 Department of Ophthalmology, Wuhan No. 1 Hospital, Wuhan 430023, Hubei Province, China; 2 Department of Ophthalmology, Wuhan Hospital of Traditional Chinese and Western Medicine, Wuhan 430023, Hubei Province, China

**Keywords:** immune checkpoint inhibitors, uveal melanoma, lactate dehydrogenase, prognosis

## Abstract

Objective: We performed the meta-analysis to explore the predictive value of lactate dehydrogenase (LDH) levels in uveal melanoma (UM) patients receiving immune checkpoint inhibitors (ICIs).

Methods: Eligible articles were obtained through EMBASE, PubMed, Google Scholar, and the Cochrane Library, until March 23, 2023. The clinical outcomes evaluated in this study encompassed overall survival (OS) and progression-free survival (PFS).

Results: This meta-analysis comprised eight studies with a combined total of 383 patients. The results showed that patients with high LDH levels had noticeably worse OS (HR: 3.445, 95% CI: 2.504-4.740, *p* < 0.001) and PFS (HR: 1.720, 95% CI: 1.429-2.070, *p* < 0.001). Subgroup analysis confirmed that the upper limit of normal was the ideal cut-off value for LDH. In multivariate analysis, we also found that high LDH levels significantly predicted shorter OS (HR: 3.405, 95% CI: 1.827-6.348, *p* < 0.001) and PFS (HR: 2.519, 95% CI: 1.557-4.076, *p* < 0.001) in UM patients. The sensitivity analysis and publication bias test supported the reliability of our results.

Conclusions: In UM patients treated with ICIs, the LDH levels were reliable indicators of prognosis.

## INTRODUCTION

The most common primary intraocular malignancy in adults is uveal melanoma (UM). UM develops from melanocytes in the iris, ciliary body, or choroid, and they exhibit distinct clinical and biological features from cutaneous melanoma [1–3]. In most cases, the primary disease can be effectively treated with radiotherapy or enucleation. However, approximately 50% of patients subsequently develop metastatic disease, which typically spreads to the liver [4–6].

Currently, immune checkpoint inhibitors (ICIs) targeting CTLA-4 and/or PD-1/PD-L1 are frequently employed to treat metastatic uveal melanoma (mUM) [7, 8]. Although ICIs have significantly improved patient prognosis for cutaneous and mucosal melanoma, mUM patients do not receive equivalent benefits. A prospective study investigated first-line pembrolizumab treatment and found that patients who achieved objective clinical benefit had a median overall survival (OS) of 12.8 months, a result consistent with other agents [9]. UM patients are typically lacking certain features that are thought to increase the likelihood of responding positively to ICIs. These features include strong PD-1 expression, a high tumor mutational burden, and no liver metastases [10–12]. As such, the identification of prognostic indicators in UM patients receiving ICIs is therefore necessary.

Lactate dehydrogenase (LDH) is a well-established prognostic marker for various advanced solid tumors, including UM [13–16]. LDH can change the tumor microenvironment by enhancing lactate generation and encouraging immunosuppression [17, 18]. Some recent studies by Waninger et al. [19] and Kelly et al. [20] revealed that high LDH levels were associated with shorter OS and PFS in mUM patients treated with ICIs, whereas Yildiz et al. [21] and Namikawa et al. [22] found that LDH levels in mUM patients were not associated with ICI therapeutic efficacy. To address the aforementioned controversy, a meta-analysis was conducted to determine the predictive significance of baseline LDH levels in UM patients who were treated with ICI. This analysis may assist in determining the prognosis and developing effective treatment strategies.

## MATERIALS AND METHODS

### Literature search strategies

The present analysis was conducted in accordance with the PRISMA statement [23]. On March 23, 2023, a thorough article search was conducted using the Cochrane Library, PubMed, and EMBASE. The search terms “Lactate dehydrogenase”, “LDH”, “Immune Checkpoint Inhibitors [Mesh]”, and “Uveal Neoplasms [Mesh]”, along with their entry terms, such as “Immune Checkpoint Blockers”, “Immune Checkpoint Blockade”, “PD-1 Inhibitors”, “PD-L1 Inhibitors”, “CTLA-4 Inhibitors”, “Pembrolizumab”, “Nivolumab”, “Atezolizumab”, “Ipilimumab”, “Avelumab”, “Tremelimumab”, “Durvalumab”, “Cemiplimab”, “Uveal Melanoma” were searched within [All Fields]. Searches are restricted to English literature. Additionally, grey literature was searched using Google Scholar, and the reference lists of eligible publications were manually retrieved. Please refer to Supplementary Table 1 for a detailed account of the search strategies.

### Inclusion and exclusion criteria

Our study included only those research articles that met the following criteria: patients with a UM diagnosis, treatment with ICIs, and evaluation of the prognostic value of the LDH. In addition, these outcomes (OS and progression-free survival (PFS)) were presented in at least one of the articles. Conference abstracts were not included. Only the publications with the most thorough data and robust methods were chosen in circumstances where research reported overlapping patients.

### Data extraction and quality assessment

We extracted various data points, including author, publication year, study region, study design, study duration, sample size, age, gender distribution, therapeutic drugs, and outcomes. In the case of univariate and multivariate analyses of HR, we prioritized the extraction of the latter [24]. The Newcastle-Ottawa Scale (NOS) score was used to estimate the quality of included studies, and we determined that high-quality literature had a score of 6 or higher [7, 13].

### Statistical methods

The statistical analysis was performed using Stata 15.0. We utilised a random effect model if *p* < 0.1 and I^2^ > 50%; otherwise, a fixed effect model was used. The Egger and Begg tests were used to estimate the degree of bias. A sensitivity analysis was also conducted, where each study was excluded independently, to assess the robustness of the results.

### Availability of data and materials

The data that support the findings of this study are available from the corresponding author upon reasonable request.

## RESULTS

### Characteristics of studies

**Figure 1:**
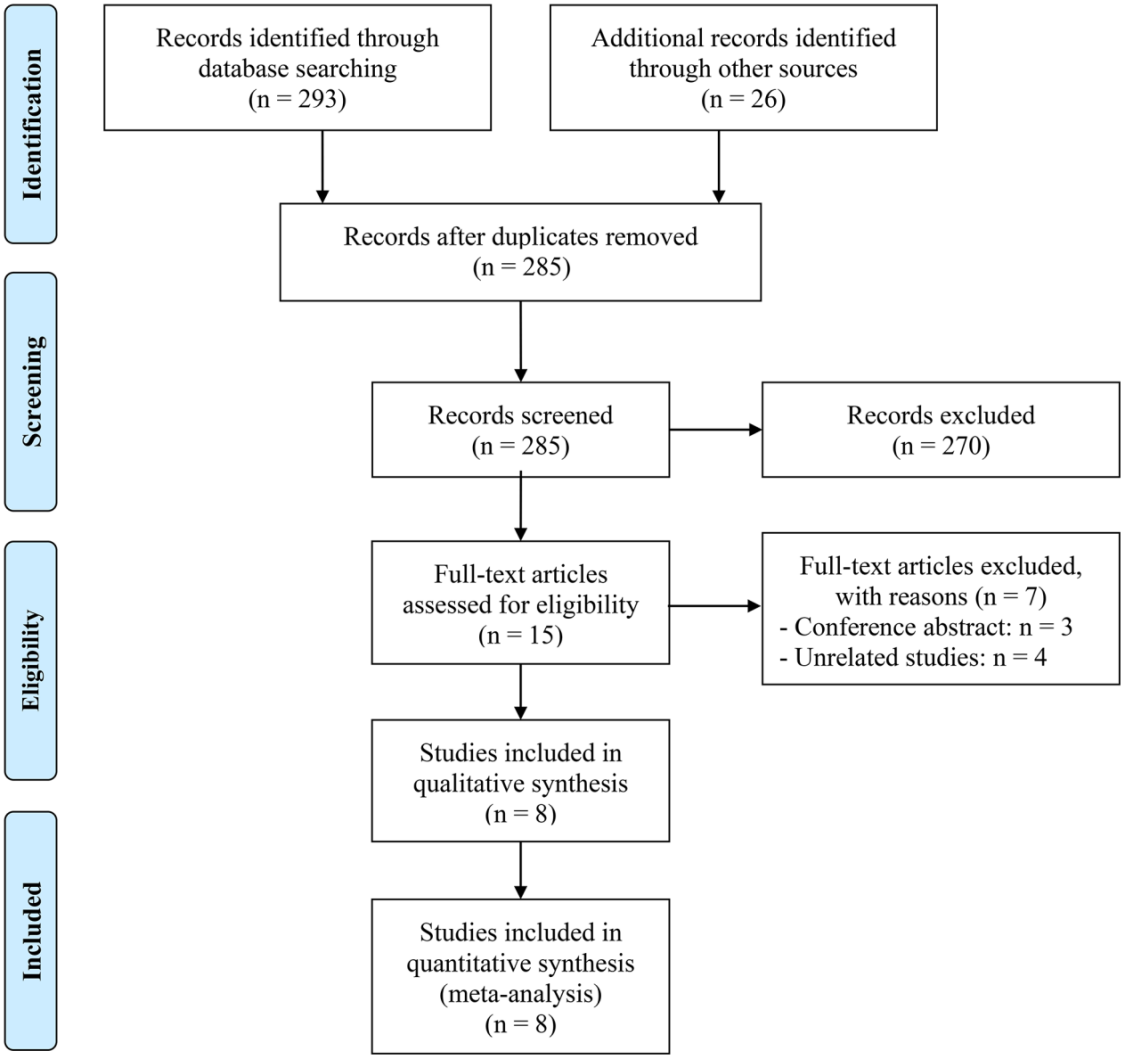
The flow diagram of identifying eligible studies.

Following the initial search, 34 duplicate studies were excluded. Subsequently, after a thorough review of the titles and abstracts, 270 articles were removed. The remaining 15 articles were then subjected to a detailed examination of their full texts. Ultimately, eight articles, comprising a total of 383 patients, were selected for inclusion in the analysis [19–22, 25–28]. Figure 1 displays the PRISMA flow diagram illustrative of the selection procedure. The primary attributes of the studies examined are outlined in Table 1. For all publications, the NOS scores ranged from 6 to 8, indicating a low probability of bias.

**Table 1:** Main characteristics of the studies included.

**Study**	**Study region**	**Study period**	**Study design**	**Sample size**	**Age**	**Gender(male/female)**	**Therapeutic drugs**	**Cancer stage**	**Cut-off of LDH**	**Outcomes**
Waninger et al. 2022 [19]	USA	09/2012-05/2022	R	46	61.8 (20.0)^d^	26/20	Ipilimumab/Nivolumab/Pembrolizumab/Nivolumab and Ipilimumab	mUM	ULN	OS, PFS
Kelly et al. 2021 [20]	Canada	01/2014-12/2019	R	75	36/39^b^	35/40	Anti-PD1/L1 alone or in combination with anti-CTLA4	mUM	1.5×ULN	OS, PFS
Ny et al. 2021 [26]	Swedish	02/2018-12/2018	S	39	70 (34-83)^a^	17/12	Pembrolizumab	mUM	ULN	OS, PFS
Piulats et al. 2021 [25]	Spain	04/2016-06/2017	S	52	59 (26-84)^a^	29/23	Nivolumab and Ipilimumab	mUM	ULN	OS, PFS
Yildiz et al. 2021 [21]	Turkey	01/2017-10/2020	R	17	60 (39–75)^a^	7/10	Nivolumab	mUM	ULN	OS, PFS
Namikawa et al. 2020 [22]	Japan	07/2014-07/2016	R	14	60 (42–74)^a^	11/3	Nivolumab	mUM	ULN	OS, PFS
Heppt et al. 2017 [27]	German	07/2016-10/2016	R	101	60/41^c^	58/43	Pembrolizumab/Nivolumab	mUM	ULN	OS, PFS
Luke et al. 2013 [28]	USA	-	R	39	61 (39-84)^a^	23/16	Ipilimumab	mUM	ULN	OS

^a^medians with ranges; ^b^≥ 65 vs. < 65; ^c^≥ 60 vs. < 60; ^d^medians (interquartile range); R, retrospective study; S, single-arm study; OS, overall survival; PFS, progression-free survival; PD-1, programmed cell death protein 1; PD-L1, programmed cell death 1 ligand 1; CTLA-4, cytotoxic T lymphocyte antigen 4; mUM, metastatic uveal melanoma; ULN, upper limit of normal; LDH, lactate Dehydrogenase.

A total of eight studies investigating metastatic uveal melanoma were incorporated, among which six were retrospective analyses and two were single-arm studies. For seven studies, the upper limit of normal LDH was used as the boundary, while for one study, the boundary was set at 1.5 times the upper limit of normal (Table 1).

### Baseline LDH levels and OS

**Figure 2:**
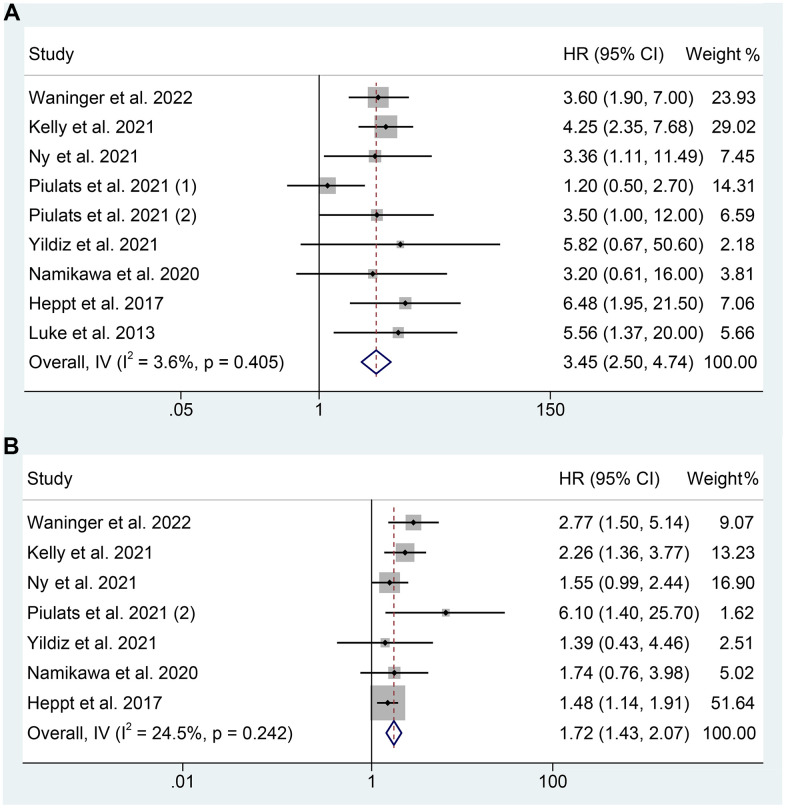
Forest plots of the relationship between baseline LDH levels and overall survival (**A**). Forest plots of the relationship between baseline LDH levels and progression-free survival (**B**). HR, hazard ratio; CL, confidence interval.

We analyzed data from 8 studies (383 patients) to investigate the correlation between LDH levels and OS in mUM patients receiving ICIs. A fixed-effects model was used due to no significant heterogeneity (I^2^ = 3.6%, *p* = 0.405), as demonstrated in Figure 2A. The findings demonstrated that patients with high LDH levels had a considerably shorter OS (HR: 3.445, 95% CI: 2.504-4.740, *p* < 0.001) than those with low LDH levels.

### Baseline LDH levels and PFS

The association between LDH levels and PFS in mUM patients receiving ICIs was examined in 7 studies comprising 344 patients. The pooled HR revealed that high LDH levels increased the risk of progression by 72% (HR: 1.720, 95% CI: 1.429-2.070, *p* < 0.001, Figure 2B). There was no significant heterogeneity observed, and a fixed effects model was employed (I^2^ = 24.5%, *p* = 0.242, Figure 2B).

### Subgroup analyses

**Figure 3:**
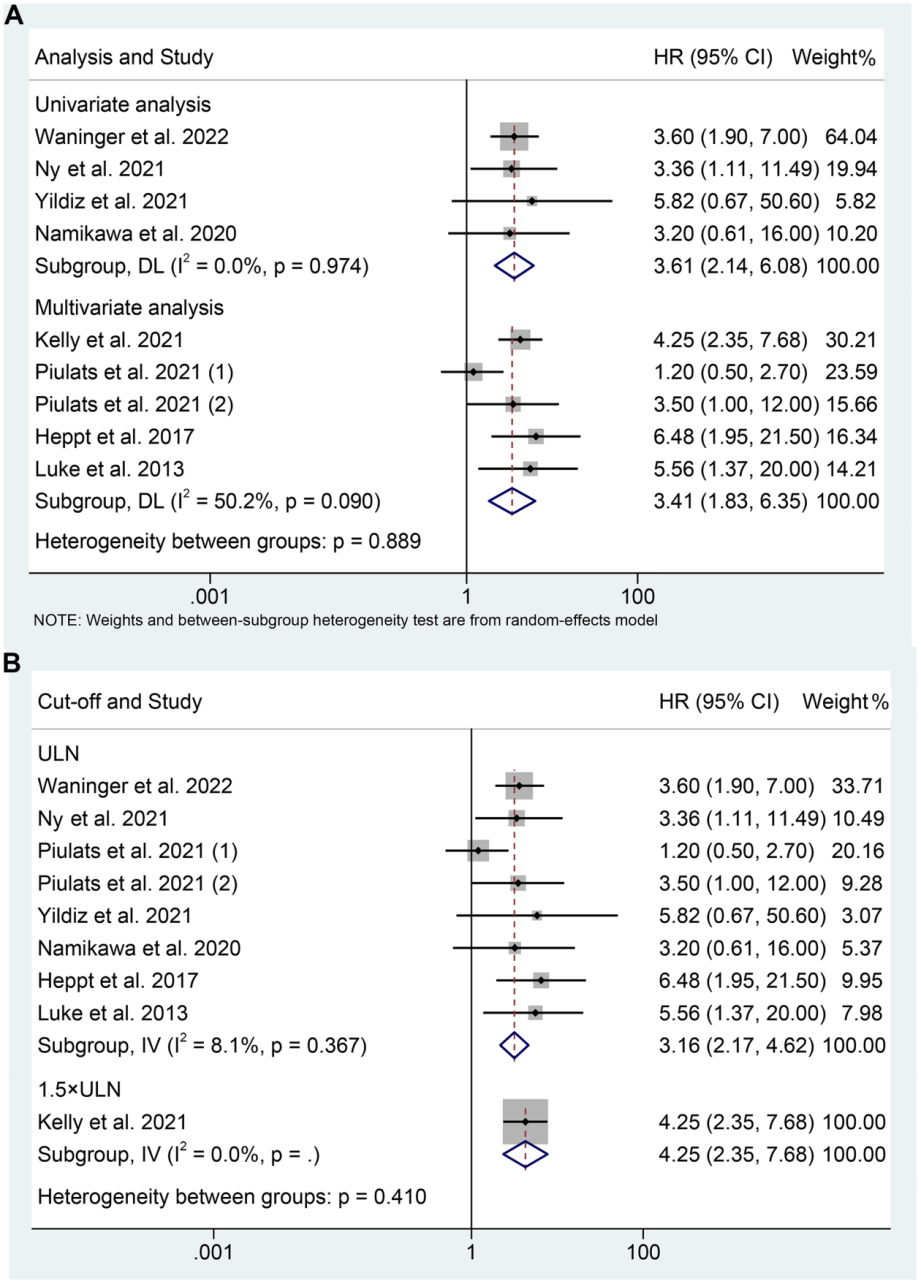
Subgroup analysis of overall survival based on analysis (**A**); Subgroup analysis of overall survival based on cut-off (**B**). HR, hazard ratio; CL, confidence interval; ULN, upper limit of normal.

We performed subgroup analyses based on the analytical method, and we discovered that mUM patients with elevated LDH levels had a shorter OS in both multivariate analyses (I^2^ = 50.2%, *p* = 0.090; HR: 3.405, 95% CI: 1.827-6.348, *p* < 0.001) and univariate analysis (I^2^ = 0%, *p* = 0.974; HR: 3.608, 95% CI: 2.141-6.079, *p* < 0.001) (Figure 3A). Differences in LDH cut-off values can significantly affect the assessment of the efficacy of ICIs in mUM patients. We performed subgroup analyses according to different LDH cutoff values. Current evidence confirms that high LDH levels at a cut-off value of the upper limit of normal significantly predicted poorer OS in mUM patients (I^2^ = 8.1%, *p* = 0.367; HR: 3.162, 95% CI: 2.165-4.617, *p* < 0.001, Figure 3B).

**Figure 4:**
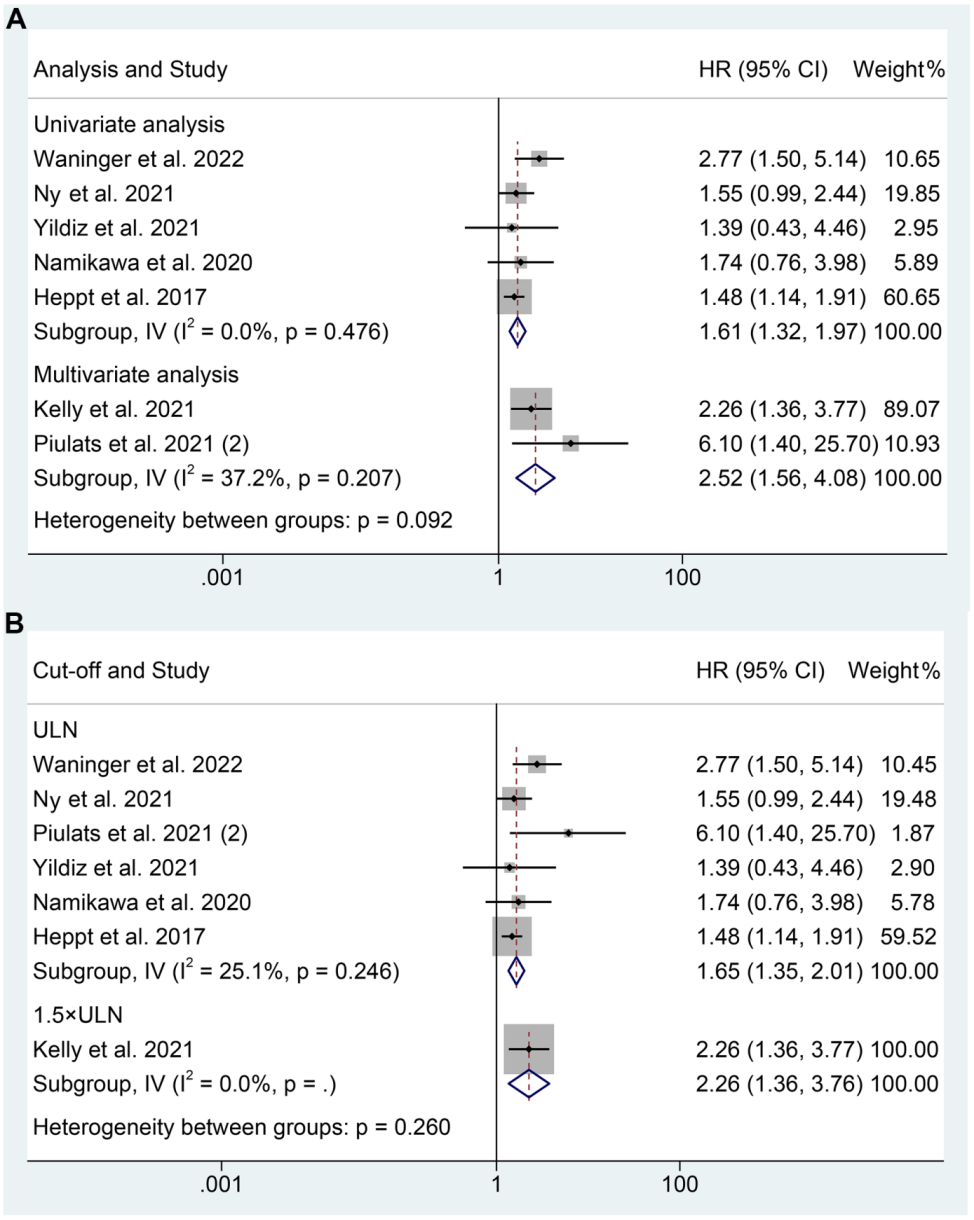
Subgroup analysis of progression-free survival based on analysis (**A**); Subgroup analysis of progression-free survival based on cut-off (**B**). HR, hazard ratio; CL, confidence interval; ULN, upper limit of normal.

As for PFS, our analysis also revealed that mUM patients with elevated LDH levels experienced a shorter OS, as demonstrated by both multivariate analysis (I^2^ = 37.2%, *p* = 0.207; HR: 2.519, 95% CI: 1.557-4.076, *p* < 0.001) and univariate analysis (I^2^ = 0%, *p* = 0.476; HR: 1.609, 95% CI: 1.316-1.967, *p* < 0.001) (Figure 4A). Besides, we found that high LDH levels at a cut-off value of the upper limit of normal were significantly associated with shorter PFS in mUM patients (I^2^ = 25.1%, *p* = 0.246; HR: 1.650, 95% CI: 1.352-2.013, *p* < 0.001, Figure 4B).

### Sensitivity analysis and publication bias

**Figure 5:**
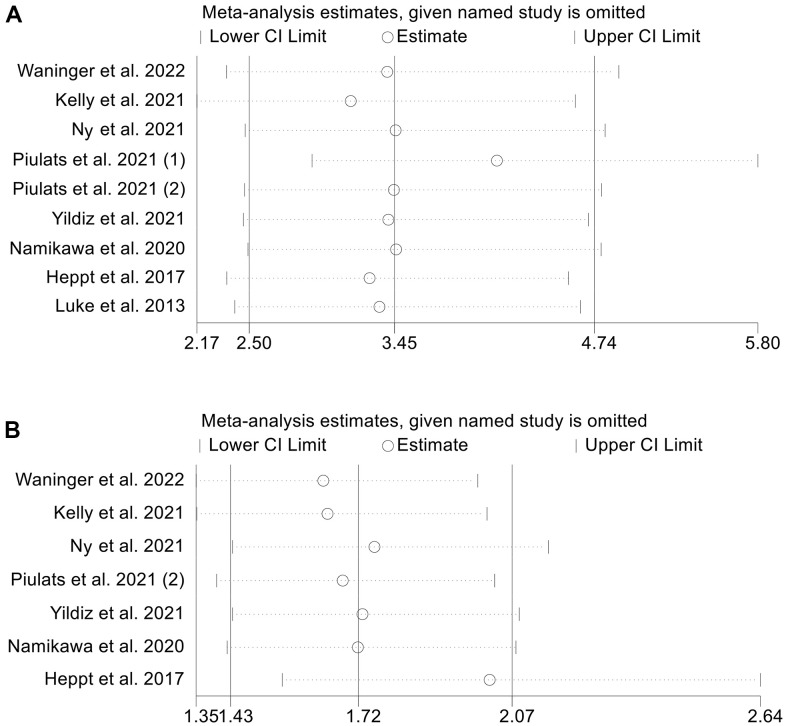
Sensitivity analysis of the association between baseline LDH levels and overall survival (**A**) and progression-free survival (**B**). CL, confidence interval.

To evaluate the potential influence of each study on the results, we employ the leave-one-out method. Our findings indicated that omitting one research at a time had no significant effect on the combined HR for OS, ranging from 3.162 (95% CI: 2.166-4.617, after removing Kelly et al. 2021) to 4.109 (95% CI: 2.911–5.799, after removing Piulats et al. 2021 (1), Figure 5A). Similar to that, the sensitivity analyses’ pooled HR for PFS did not show any significant differences, with a pooled HR ranging from 1.640 (95% CI: 1.350-1.992, after removing Waninger et al. 2022) to 2.019 (95% CI: 1.547-2.636, after removing Heppt et al. 2017, Figure 5B). These results indicate that our findings are robust and reliable.

To measure publication bias in the meta-analysis, we used Begg’s and Egger’s tests. The results revealed that there was no significant publication bias in OS (Egger’s test: *P* = 0.705, Begg’s test: *P* = 0.917) and PFS (Egger’s test: *P* = 0.120, Begg’s test: *P* = 0.133).

## DISCUSSION

We aimed to investigate the predictive value of LDH in UM patients, and the pooled data unequivocally revealed a significant correlation between higher LDH levels and shorter OS and PFS. In addition, these findings remained consistent following sensitivity analysis and subgroup analysis. This represents the first comprehensive meta-analysis examining the influence of LDH on the prognosis of UM patients treated with ICIs. Since LDH is a readily available clinical parameter, evaluating it prior to ICI treatment can aid physicians in predicting clinical outcomes more accurately and efficiently. This information can be used to promptly adjust treatment, thereby further increasing the benefit rates.

Neoplastic cells frequently display altered metabolism, characterized by heightened glucose uptake and increased lactate synthesis, even in the presence of oxygen [29]. The Warburg effect is a phenomenon that is one of the basic metabolic rewiring processes that take place throughout cancer transformation [30]. Initially, it was believed that this phenomenon occurred due to mitochondrial dysfunction. However, it is now understood that cancer cells rely on various glucose metabolites for the synthesis of nucleic acids, fatty acids, and lactate. This dependency is crucial for intracellular signalling, microenvironmental angiogenesis, and overall tumor growth [31]. LDH-A and LDH-B are the two primary subunits that make up LDH, a crucial enzyme in the glycolytic process [32]. This tetrameric enzyme catalyzes the last step of glycolysis by converting pyruvate to lactate while also oxidizing nicotinamide adenine dinucleotide dehydrogenase (NADH) to NAD^+^.

In addition to playing a critical role in cancer metabolism, LDH increase also alters the tumour microenvironment, which allows neoplastic cells to avoid the immune system and worsen prognosis [13–16, 18, 33]. Increased lactate production caused by LDH-A changes the tumour microenvironment by promoting immune-suppressive cells like tumor-associated macrophages, myeloid-derived suppressor cells, and dendritic cells while inhibiting cytotoxic cells like cytotoxic T lymphocytes and natural killer cells [18, 33, 34]. This immune suppression caused by LDH-A leads to resistance to chemo/radio/targeted therapy [18, 33, 35, 36]. The prognostic value of LDH in cutaneous melanoma has been firmly established and is now incorporated into the AJCC staging system [37]. Our study confirmed that elevated LDH may increase the resistance to ICIs in mUM patients through these mechanisms mentioned above.

However, it is worth noting that there are insufficient data to support our analysis of the relationship between LDH levels and objective response rates and complications in patients with mUM treated with ICIs. In addition, although eight studies were included in this study, the number of populations included was not very large. Henceforth, it is imperative to obtain more high-quality studies with equivalent sample sizes to corroborate and augment our inferences.

## Supplementary Material

Supplementary Table 1
