# Supplementary material for: Prognostic value of lactate dehydrogenase in patients with uveal melanoma treated with immune checkpoint inhibition
Source: Aging (Albany NY). 2023 Sep 5;15(17):8770–81. doi: 10.18632/aging.204996 (PMC10522394; doi:10.18632/aging.204996)
Supplement: Supplementary Table 1 [file aging-15-204996-s001.pdf]

SUPPLEMENTARY TABLE

Supplementary Table 1. The detailed search strategies for Pubmed.

(((((((Uveal Neoplasms) OR (Uveal Neoplasm)) OR (Melanoma of the Uveal)) OR (Melanoma, Uveal)) OR (Uveal Melanoma)) OR (Uveal Neoplasms[MeSH Terms]))) AND (((Lactate dehydrogenase[Title/Abstract]) OR (LDH[Title/Abstract])))) AND (((Immune Checkpoint Inhibitors) OR (Checkpoint Inhibitors, Immune)) OR (Immune Checkpoint Inhibitor)) OR (Checkpoint Inhibitor, Immune)) OR (Immune Checkpoint Blockers)) OR (Checkpoint Blockers, Immune)) OR (Immune Checkpoint Blockade)) OR (Checkpoint Blockade, Immune)) OR (Immune Checkpoint Inhibition)) OR (Checkpoint Inhibition, Immune)) OR (PD-L1 Inhibitors)) OR (PD L1 Inhibitors)) OR (PD-L1 Inhibitor)) OR (PD L1 Inhibitor)) OR (Programmed Death-Ligand 1 Inhibitors)) OR (Programmed Death Ligand 1 Inhibitors)) OR (PD-1-PD-L1 Blockade)) OR (Blockade, PD-1-PD-L1)) OR (PD 1 PD L1 Blockade)) OR (CTLA-4 Inhibitors)) OR (CTLA 4 Inhibitors)) OR (CTLA-4 Inhibitor)) OR (CTLA 4 Inhibitor)) OR (Cytotoxic T-Lymphocyte-Associated Protein 4 Inhibitors)) OR (Cytotoxic T Lymphocyte Associated Protein 4 Inhibitors)) OR (Cytotoxic T-Lymphocyte-Associated Protein 4 Inhibitor)) OR (Cytotoxic T Lymphocyte Associated Protein 4 Inhibitor)) OR (PD-1 Inhibitors)) OR (PD-1 Inhibitor)) OR (PD 1 Inhibitors)) OR (Inhibitor, PD-1)) OR (PD 1 Inhibitor)) OR (Programmed Cell Death Protein 1 Inhibitor)) OR (Programmed Cell Death Protein 1 Inhibitors)) OR ("Immune Checkpoint Inhibitors"[Mesh])))))))) OR (pembrolizumab)) OR (nivolumab)) OR (atezolizumab)) OR (ipilimumab)) OR (avelumab)) OR (tremelimumab)) OR (durvalumab)) OR (cemiplimab))) OR (anti-PD-1 antibodies)) OR (anti-PD-1 antibody))) OR (anti-PD-L1 antibody)) OR (anti-PD-L1 antibodies)))).
